# The intriguing effect of ethanol and nicotine on acetylcholine-sensitive potassium current *I*_KAch_: Insight from a quantitative model

**DOI:** 10.1371/journal.pone.0223448

**Published:** 2019-10-10

**Authors:** Jiří Šimurda, Milena Šimurdová, Markéta Bébarová

**Affiliations:** Department of Physiology, Faculty of Medicine, Masaryk University, Kamenice, Brno, Czech Republic; University at Buffalo - The State University of New York, UNITED STATES

## Abstract

Recent experimental work has revealed unusual features of the effect of certain drugs on cardiac inwardly rectifying potassium currents, including the constitutively active and acetylcholine-induced components of acetylcholine-sensitive current (*I*_KAch_). These unusual features have included alternating susceptibility of the current components to activation and inhibition induced by ethanol or nicotine applied at various concentrations, and significant correlation between the drug effect and the current magnitude measured under drug-free conditions. To explain these complex drug effects, we have developed a new type of quantitative model to offer a possible interpretation of the effect of ethanol and nicotine on the *I*_KAch_ channels. The model is based on a description of *I*_KAch_ as a sum of particular currents related to the populations of channels formed by identical assemblies of different α-subunits. Assuming two different channel populations in agreement with the two reported functional *I*_KAch_-channels (GIRK1/4 and GIRK4), the model was able to simulate all the above-mentioned characteristic features of drug-channel interactions and also the dispersion of the current measured in different cells. The formulation of our model equations allows the model to be incorporated easily into the existing integrative models of electrical activity of cardiac cells involving quantitative description of *I*_KAch_. We suppose that the model could also help make sense of certain observations related to the channels that do not show inward rectification. This new ionic channel model, based on a concept we call *population type*, may allow for the interpretation of complex interactions of drugs with ionic channels of various types, which cannot be done using the ionic channel models available so far.

## Introduction

Our recent experimental work has revealed unusual features of the effect of ethanol and nicotine on cardiac ionic membrane channels responsible for inwardly rectifying potassium currents (*I*_Kir_); both drugs exhibited inhibition or activation depending on drug concentration. Furthermore, the drug effect correlated with the current magnitude measured under drug-free conditions [1–4]. A similar dualistic concentration-dependent characteristic was previously demonstrated in the effect of halothane on GIRK channels expressed in *Xenopus laevis* oocytes [5–6].

In an attempt to explain these drug effects, we designed a quantitative model considering the known structure of the inwardly rectifying potassium channels, which are composed of various homomeric or heteromeric assemblies of channel subunits (Kir2.x, Kir3.x, and Kir6.x/SURx) with a considerable diversity in regional expression of the underlying subunits [7] (for reviews, see [8–9]). Different combinations of the expressed subunits result in specific functional properties of the measured currents, including drug-channel interactions.

To our knowledge, the existing integral mathematical models of the inwardly rectifying potassium currents ignore the fact that these currents can be seen as the sum of separate currents. In our model, the Kir currents are described as a total of *n* particular independent currents *I*_Kir,x,j_ via individual populations (*j*) of identical channels, i.e. channels formed by the same combination of subunits. The number *n* of constituents (each created by identical channels) is unknown. To describe the effect of ethanol on *I*_K1_ in ventricular and atrial cardiomyocytes [1–2], we tentatively introduced a minimum number (*n* = 3) needed to simulate the available observations [10]. Three α-subunits (Kir2.1, Kir2.2, and Kir2.3) are known to participate in the formation of *I*_K1_ channels (for review, see [9]).

In this study, we apply the conception of different channel populations to analyse the responses of acetylcholine-sensitive inward rectifying potassium current (*I*_KAch_) to ethanol [3] and nicotine [4]. These responses showed some similar features to those revealed previously in the ethanol-*I*_K1_ interactions [1–2], as mentioned above. In the current version of the model, we considered two different populations of the *I*_KAch_ channels (*n* = 2), because only two combinations of α-subunit assemblies, namely heterotetramers Kir3.1/ Kir3.4 (GIRK1/4) and homotetramers Kir3.4 (GIRK4), have been identified in cardiac atrial cells [11–18] (for review, see [9]). Homotetramers GIRK1 appeared to be nonconductive [14, 19]. The proposed quantitative model offers a possible mechanism for the effect of ethanol and nicotine on two components of *I*_KAch_, namely the constitutively active (*I*_KAch_CONST_) and the acetylcholine-induced (*I*_KAch_ACH_).

## Results

### Kir channel populations—Model description

In the model described in our previous study [10], Kir currents (often recalculated to current densities in pA/pF) were generally expressed as:

$$I=\sum_{j=1}^{n}f_{j}I_{j}=\sum_{j=1}^{n}f_{j}G_{j}(U-U_{K}),\;(\sum_{j=1}^{n}f_{j}=1).$$ (1)

In this equation, *n* denotes a number of independent components *I*_j_ of the current *I* (which are related to individual channel populations formed by different identical assemblies of α-subunits), *f*_j_ denotes fraction of the *j*^th^ identical channel population, and *U*, *U*_K_, and *G*_j_ denote membrane voltage, equilibrium voltage for potassium ions, and fractional conductivities, respectively. This concept was a useful tool for describing complex interactions of *I*_K1_ (Kir2.x) with ethanol [10]. As mentioned above, a minimum number *n* = 3 was selected to enable reproduction of available experimental results and to keep the model as simple as possible.

In the case of *I*_KAch_, we confined the number of channel populations to *n* = 2 because GIRK4 homotetramers and GIRK1/4 heterotetramers are the only functional assemblies underlying *I*_KAch_ in cardiac cells (for review, see [18]). Significant differences occur between GIRK4 and GIRK1/4 channels (e.g. different sensitivity to [Na^+^]_i_), although both tetramers share ~44% sequence identity [16]. The two components of *I*_KAch_ (*I*_KAch_CONST_ and *I*_KAch_ACH_) were described by the same equations, but with different numeric values for the parameters.

Previous studies have shown that a hydrophobic *pocket* in the cytoplasmic domain is a putative activating binding site for alcohols and other substances [20–22]. The pocket may be occupied by one or more ethanol molecules, indicating a sequential bond. Alternate sites were shown to be responsible for inhibition [20]. As little is known about the underlying mechanisms, we used the simple description of inhibition to display experimental results.

**Fig 1:**
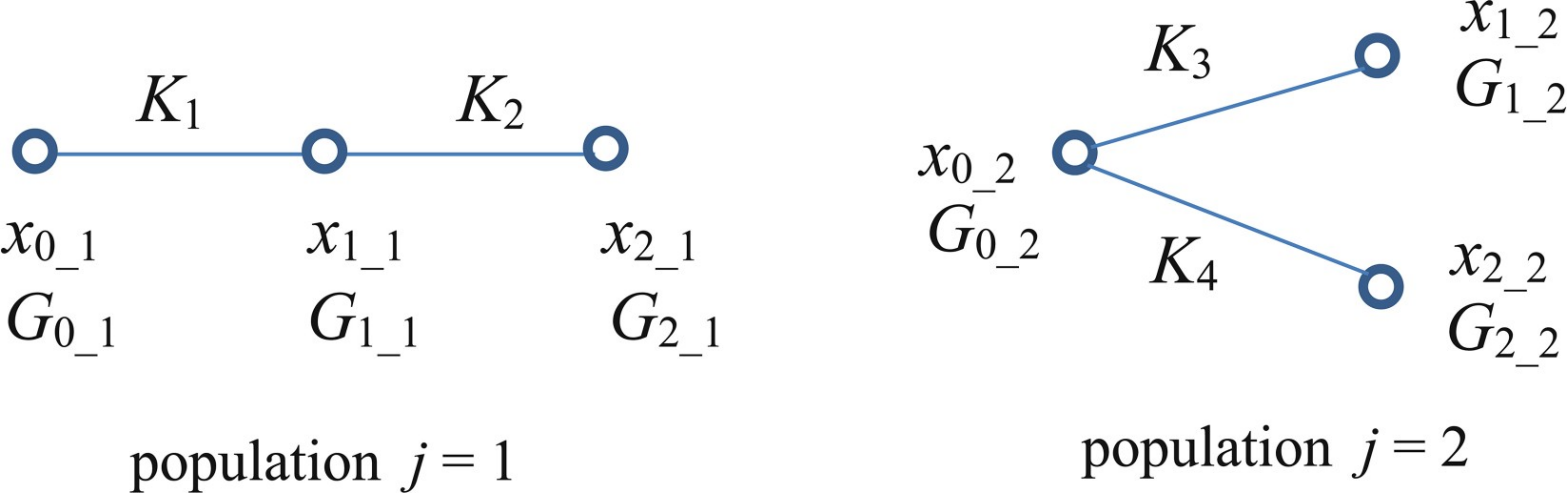
A schematic representation of the drug-channel interactions. One or two drug molecules can be bound in the pocket related to activation (left). One drug molecule can be bound to one of two binding sites related to inhibition (right). *K*_1_, *K*_2,_ *K*_3_, *K*4—dissociation constants; *x*k_j—probabilities of channels to be found in state *k* (*k* = 0, 1, or 2) pertaining to *j*^th^ channel population (*j* = 1 or 2); *G*_k_1,_ *G*k_2—partial steady-state conductivities of the 1^st^ and 2^nd^ channel population related to the drug occupation of binding sites.

Both populations (*f*_1_, *f*_2_) may include activation and inhibition binding sites, one of which is likely to dominate. However, as the simplest way to comply with the described concept, we introduced the putative pocket related to activation to the first population (*j* = 1) of identical channels. Two additional binding sites related to inhibition were assigned to the second population (*j* = 2), as illustrated in Fig 1.

The symbols *x*_0_1_ and *x*_0_2_ in Fig 1 denote probabilities of the respective channel population to be found drug-free. The probabilities that channels belonging to population *j* = 1 are occupied by one or two drug molecules are designated *x*_1_1_, *x*_2_1_. Probabilities that the first or the second binding site in channels of population *j* = 2 are occupied by a drug molecule are designated *x*_1_2_ and *x*_2_2_, respectively. If all channels of the given population were found in one of the three states shown in the schemes above, the conductivity of this population at a given membrane voltage *U* would take one of the corresponding values *G*_0_1_, *G*_1_1_, *G*_2_1_, or *G*_0_2_, *G*_1_2_, *G*_2_2_.

Transient changes of Kir currents evoked by application of ethanol (and some other drugs) appeared to be slow, lasting up to 10^2^ seconds [1–2, 23]. If we assume (in line with Šimurda *et al*. [10]) that the drug binding velocity is much higher than the subsequent (allosteric) conformational changes governing channel conductivities, then the probabilities *x*_k_j_ (*k* refers to occupation of binding sites, *j* to populations of identical channels) may be assumed to keep their steady state values depending only on the drug concentration *c* and the dissociation constants *K*_1_, *K*_2_, *K*_3_ and *K*_4_. In this case, *x*_k_j_ can be expressed as solutions of linear algebraic equations related to the above schemes describing drug-binding in the channel population 1 and 2.

$$x_{{}_{0\_1}}=\frac{1}{1+\frac{c}{K_{1}}+\frac{c^{2}}{K_{1}K_{2}{}^{}}},\;x_{{}_{1\_1}}=x_{{}_{0\_1}}\frac{c}{K_{1}},\;x_{{}_{2\_1}}=x_{{}_{0\_1}}\frac{c^{2}}{K_{1}K_{2}},$$ (2A)

$$x_{{}_{0\_2}}=\frac{1}{1+\frac{c}{K_{3}}+\frac{c^{}}{K_{4}^{}}},\;x_{{}_{1\_2}}=x_{{}_{0\_2}}\frac{c}{K_{3}},\;x_{{}_{2\_2}}=x_{{}_{0\_2}}\frac{c}{K_{4}^{}}.$$ (2B)

Evidently,

$$x_{0\_1}+x_{1\_1}+x_{2\_1}=1,\;x_{0\_2}+x_{1\_2}+x_{2\_2}=1.$$

Conductivities of the first and the second population (*G*_1_ and *G*_2_, respectively) under steady state conditions may then be expressed as

$$G_{1}=x_{0\_1}G_{0\_1}+x_{1\_1}G_{1\_1}+x_{2\_1}G_{2\_1},\;G_{2}=x_{0\_2}G_{0\_2}+x_{1\_2}G_{1\_2}+x_{2\_2}G_{2\_2},$$ (3)

and in agreement with (1)

$$I=(f_{1}G_{1}+f_{2}G_{2})(U-U_{K}).$$ (4)

Under drug-free conditions (c = 0),

$$I_{0}=(f_{1}G_{0\_1}+f_{2}G_{0\_2})(U-U_{K}).$$ (5)

The conductivities are generally voltage dependent. If, however, the drug does not interfere with the structures responsible for inward rectification, the drug effect alone is voltage independent, and all conductivities may be regarded as equally dependent on the membrane voltage. The ratio

$$F=\frac{f_{1}G_{1}+f_{2}G_{2}}{f_{1}G_{0\_1}+f_{2}G_{0-2}}=\frac{I}{I_{0}}$$ (6)

may then be interpreted as a voltage-independent indicator of the drug effect. In this case, we can express all the above-defined conductivities as products of voltage-independent dimensionless parameters *h*_k_j_ and a common voltage-dependent conductivity *g*(*U*) describing inward rectification:

$$G_{k\_j}=h_{k\_j}\;g(U),$$ (7A)

$$g(U)=0.8\left(\frac{0.325}{1+e^{\frac{U+80}{5}}}+\frac{3}{1+e^{\frac{U+150}{52}}}\right).$$ (7B)

As mentioned above, the subscripts *k =* 0, 1 or 2 refer to states differing by a drug occupation of binding sites while *j* = 1 or 2 refer to populations of the identical channels. The function *g*(*U*) was obtained as a formal mathematical expression (in μS) from the current-voltage relationship, as recorded under control conditions [3].

In this study, the conductivities under the effect of the drug were regarded as equilibrated. The experimental data related to *I*_K1_ [1–2] also included transient changes of the current in response to the onset of ethanol. Corresponding transients in conductivities were described in ref. [10] by differential equations. Unfortunately, similar transients of *I*_KAch_ following drug application were not available. For possible future use, the corresponding equations are given in the Discussion.

### Effect of ethanol on *I*_KAch_

**Fig 2:**
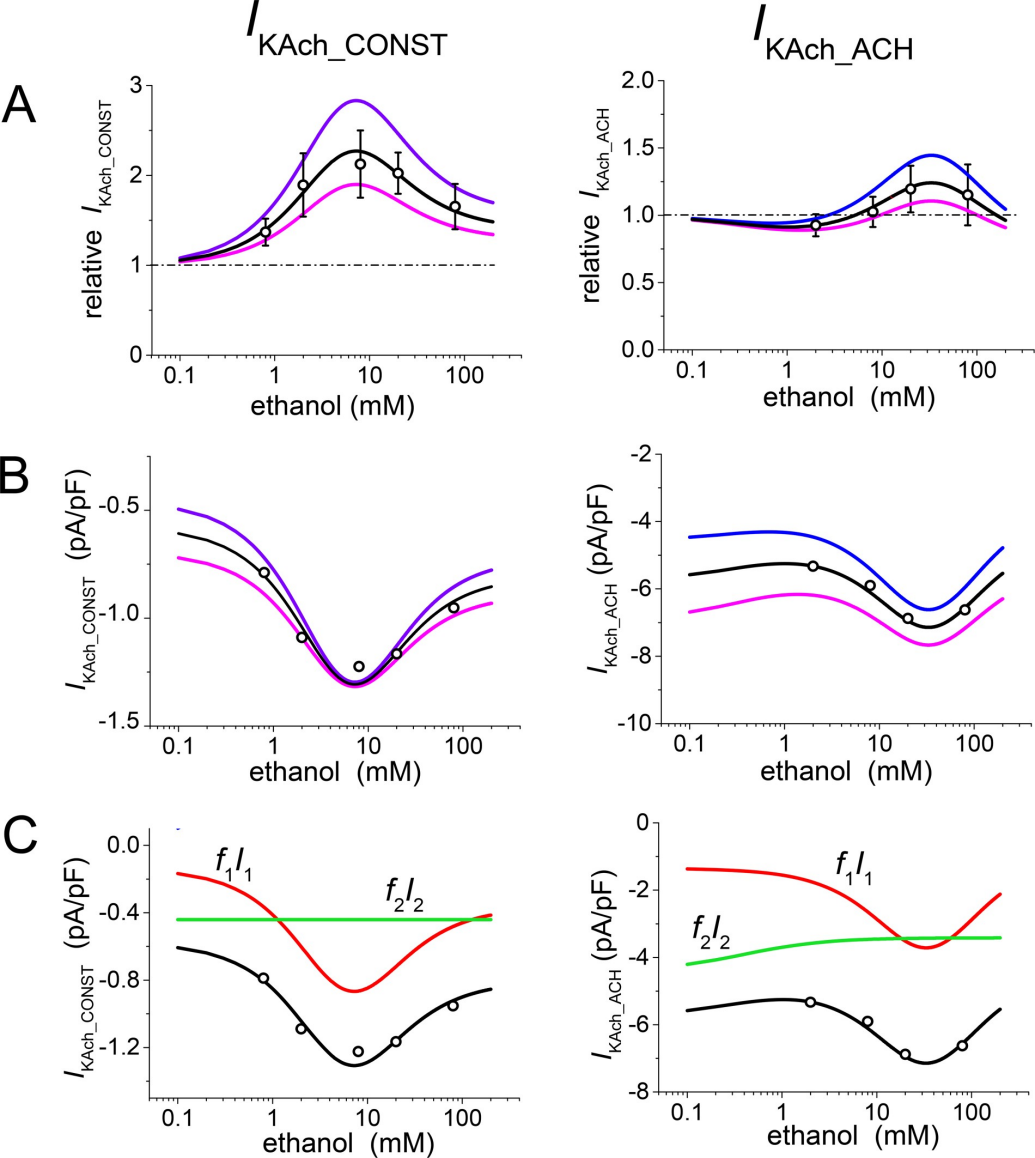
Analysis of the steady-state concentration dependence of the effect of ethanol on the constitutively active (*I*_KAch_CONST_) and acetylcholine-induced (*I*_KAch_ACH_) currents. Values of the parameters as summarized in Table 1 were used for calculations except for variations of *f*_j_ needed to simulate dispersion of the measured currents. **A**: Relative changes of *I*_KAch_CONST_ and *I*_KAch_ACH_ as functions of ethanol concentration; black lines–model; circles–experimental data (± SE according to [3]; 4–10 cells in the tested concentrations for *I*_KAch_CONST_ and 6–15 cells in the tested concentrations for *I*_KAch_ACH_). The data are related to the current densities at zero ethanol concentration. Simulations of the data dispersion result from small variations Δ*f* = 0.1 in fractions of individual populations of the *I*_KAch_ channels (*f*_1_+Δ*f*–blue lines, *f*_1_-Δ*f*–violet lines). **B:** Simulated absolute values of *I*_KAch_ resulting from multiplication of the relative values (in part A) by the current densities at zero ethanol concentration (*I*_0_, Eq (5)). **C:** Contributions of the presumptive two current constituents (red and green lines) to the resulting ethanol concentration dependence of *I*_KAch_ at the basal setting of parameters according to Eq (4); *I*_*1*_ = *G*_1_ (*U-U*_K_), *I*_*2*_ = *G*_2_ (*U-U*_K_).

The concentration dependences of the effect of ethanol on *I*_KAch_CONST_ and *I*_KAch_ACH_ are illustrated in Fig 2. The experimental results as obtained on rat atrial cardiomyocytes [3] are simulated by the solution of the model equations with the numeric values of the model parameters summarized in Table 1. The model parameters were set by repeated attempts to assure good fit with available experimental data. The number of different populations were confined to *n* = 2 in agreement with the current knowledge of the structure of the *I*_KAch_ channels. The values of parameters (Table 1) remained unaltered in all simulations relating to the effect of ethanol, except for simulations of those experimental results that were interpreted as resulting from dispersion in fractions of the individual channel populations. Table 1 shows the mean values of the fraction *f*_1_ (while *f*_2_ = 1- *f*_1_). The results of simulations related to the dispersion of the channel populations were obtained by replacing *f*_1_ with *f*_1_ + Δ*f*. The Δ*f* values are given in the figure legends.

**Table 1:** Parameters of the model for simulations of the ethanol effect on *I*_KAch_.

Current	*f*_1_ -	*K*_1_ = *K*_2_ [mM]	*K*_3_ [mM]	*K*_4_ [mM]	*h*_0_1_ -	*h*_1_1_ -	*h*_2_1_ -	*h*_0_2_ -	*h*_1_2_ -	*h*_2_2_ -
*I*_KAch_CONST_	0.68	6	0.5	150	0.013	0.2	0.035	0.09	0.09	0.09
*I*_KAch_ACH_	0.68	36	0.4	150	0.13	0.85	0.09	0.9	0.7	0.05

*f*_1_ *–*mean value of the first fraction of identical channel populations (*f*_2_ = 1- *f*_1_); *K*_1_, *K*_2,_ *K*_3_, *K*4 –drug dissociation constants; *h*_k_j_−dimensionless parameters related to the steady-state conductivities of the *j*^th^ channel population; *k* refers to occupation of binding sites.

Fig 2A shows the steady-state concentration dependence of the effect of ethanol on *I*_KAch_CONST_ (left) and *I*_KAch_ACH_ (right) in a relative scale. The quantity *F* = *I/I*_0_ (6) is plotted against ethanol concentration; simulated results (black lines) are compared with experimental data from ref. [3] (circles ± SE). The blue and violet lines show how the model allows for dispersion of the measured values. Individual cells are assumed to exhibit dispersion due to various representations of the *I*_KAch_-channel populations, which is expressed in the model as redistribution between the fractions *f*_1_ and *f*_2_. Changes in the concentration dependence induced by 10% of the channels shifting between *f*_1_ (channels showing activation) and *f*_2_ (channels showing inhibition) are plotted. Blue lines correspond to *f*_1_ = 0.68 + 0.1 and *f*_2_ = 0.32–0.1; violet lines to *f*_1_ = 0.68–0.1 and *f*_2_ = 0.32 + 0.1.

The model enables us to transform currents from the relative to the absolute scale (Fig 2B) by multiplying the relative current values by the corresponding current densities at zero ethanol concentration (*I* = *F I*_0_ according to Eq (6)). Any redistribution of the channels between *f*_1_ and *f*_2_ is accompanied by a change in the control current density (approximately the values at the lowest concentration in Fig 2B), as a consequence of differences in the conductivities *G*_0_1_ and *G*_0_2_ of the individual channel populations. The marked dispersion of the current *I* at low drug concentrations in absolute values (Fig 2B) is reflected in an increased dispersion at higher concentrations after normalization to *I*_0_ (Fig 2A).

In Fig 2C, the mean ethanol concentration dependences of both components of *I*_KAch_ (black lines in Fig 2B) are expressed as total of their presumptive constituents related to the individual channel populations (red and green lines).

**Fig 3:**
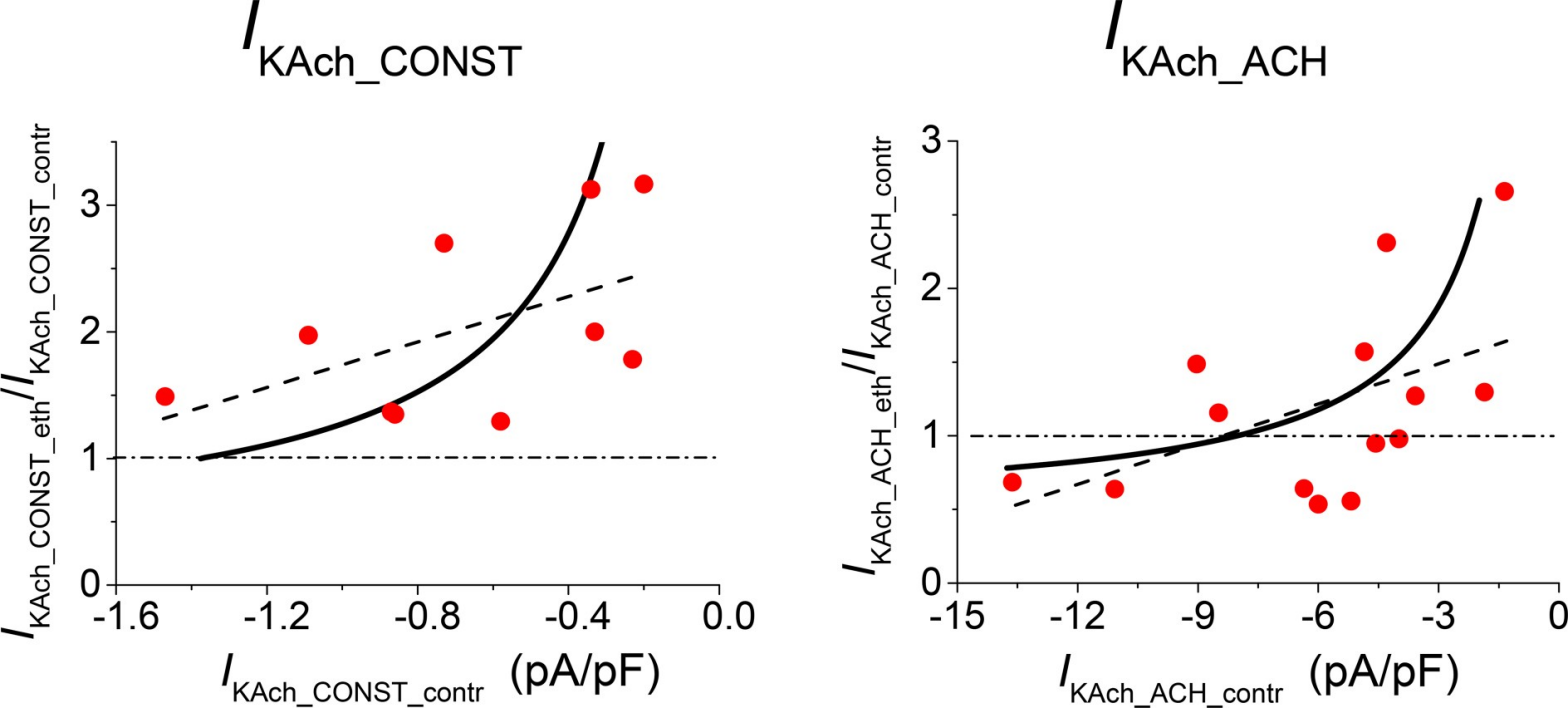
Correlation between the relative effect of ethanol (eth) and the current density of *I*_KAch_ components under ethanol-free conditions (contr). Experimental results from individual cells at an ethanol concentration of 20 mM (circles) were adopted from the original set of data from Bébarová *et al*. [3] (9 cells for *I*_KAch_CONST_ and 14 cells for *I*_KAch_ACH_). The full lines result from the model simulations with values of parameters as stated in Table 1. Dashed lines result from the linear regression analysis of the experimental data; the correlation coefficient was significant (*P* < 0.05) in *I*_KAch_ACH_. The variations of the current in control were simulated by continuous redistribution between channel populations (fractions *f*_1_ and *f*_2_ = 1- *f*_1_).

Another peculiarity of the effect of ethanol on both components of *I*_KAch_ was the correlation between the drug effect (inhibition or activation) and the current density in the absence of ethanol [3]. A similar property was observed and simulated in the interaction of *I*_K1_ with ethanol in ventricular and atrial cardiomyocytes [1–2, 10]. The solution of the model equations appeared to reproduce the drug effect as a nonlinear function of the current density in the absence of ethanol (Fig 3, full lines). The results of the model are compared in Fig 3 with experimental data from different cells (filled symbols) and with the results of linear regression (dashed lines). The simulated variations of the current in the control (*I*_KAch_CONST_contr_ and *I*_KAch_ACH_contr_) resulted from continuous redistribution between fractions *f*_1_ and *f*_2_. The model offers a prospective explanation of the correlation as a consequence of different conductivities of the channel populations and their different sensitivities to ethanol.

In the current form of the model, the drug effect itself is regarded as voltage-independent so that all the parameters in Table 1 are constants. Under this assumption, the model is restricted to a description of the effects of drugs which do not considerably interfere with the structures responsible for the non-linear current-voltage relationship (inward rectification). Otherwise, the parameters are to be regarded as voltage dependent.

**Fig 4:**
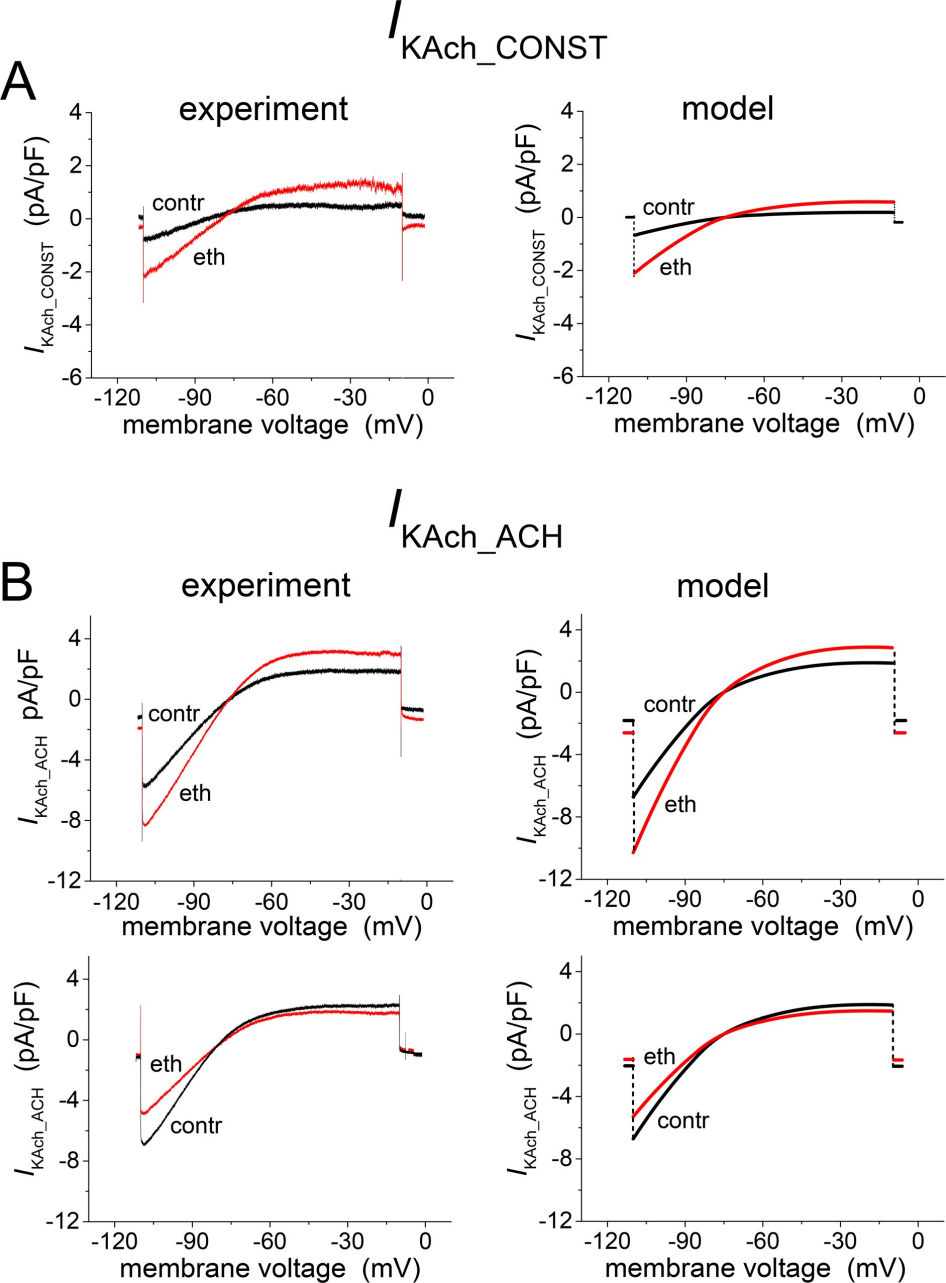
Effect of ethanol (20 mM) on the current-voltage relation of *I*_KAch_ components. Experimental (left panels; 4 cells for *I*_KAch_CONST_; 14 cells for *I*_KAch_ACH_—7 cells for the activation and 7 cells for the inhibition) and simulated (right panels) data. Activation and inhibition were simulated by redistribution of the channels between fractions *f*_1_ and *f*_2_: (*f*_1_→ *f*_1_+Δ*f*, *f*_2_→ *f*_2_-Δ*f*). **A:** Simulation (right panel; Δ*f* = 0.19) of a representative record of *I*_KAch_CONST_ (left panel). **B:** Simulations (right panels) of representative records of *I*_KAch_ACH_ (left panels) showing the ethanol-induced activation (upper panels, Δ*f* = 0.15) and the inhibition (lower panels, Δ*f* = -0.6). To better simulate the illustrated representative experimental records, the conductivity *g*(*U*) was multiplied by 0.5.

The experimental current-voltage relations of *I*_KAch_CONST_ and *I*_KAch_ACH_ in the absence and presence of ethanol (see Fig 4A and 4B in ref. [3]) were simulated by evaluation of the expressions of *I* and *I*_0_ from Eqs (4) and (5). In Fig 4 the experimental and the model results are compared. The differences between the cells showing activation and those showing inhibition of *I*_KAch_ACH_ (Fig 4B) are interpreted and simulated as a consequence of differences between the fractions *f*_1_ and *f*_2_ = 1- *f*_1_ of channel populations in individual cells.

### Effect of nicotine on *I*_KAch_

**Fig 5:**
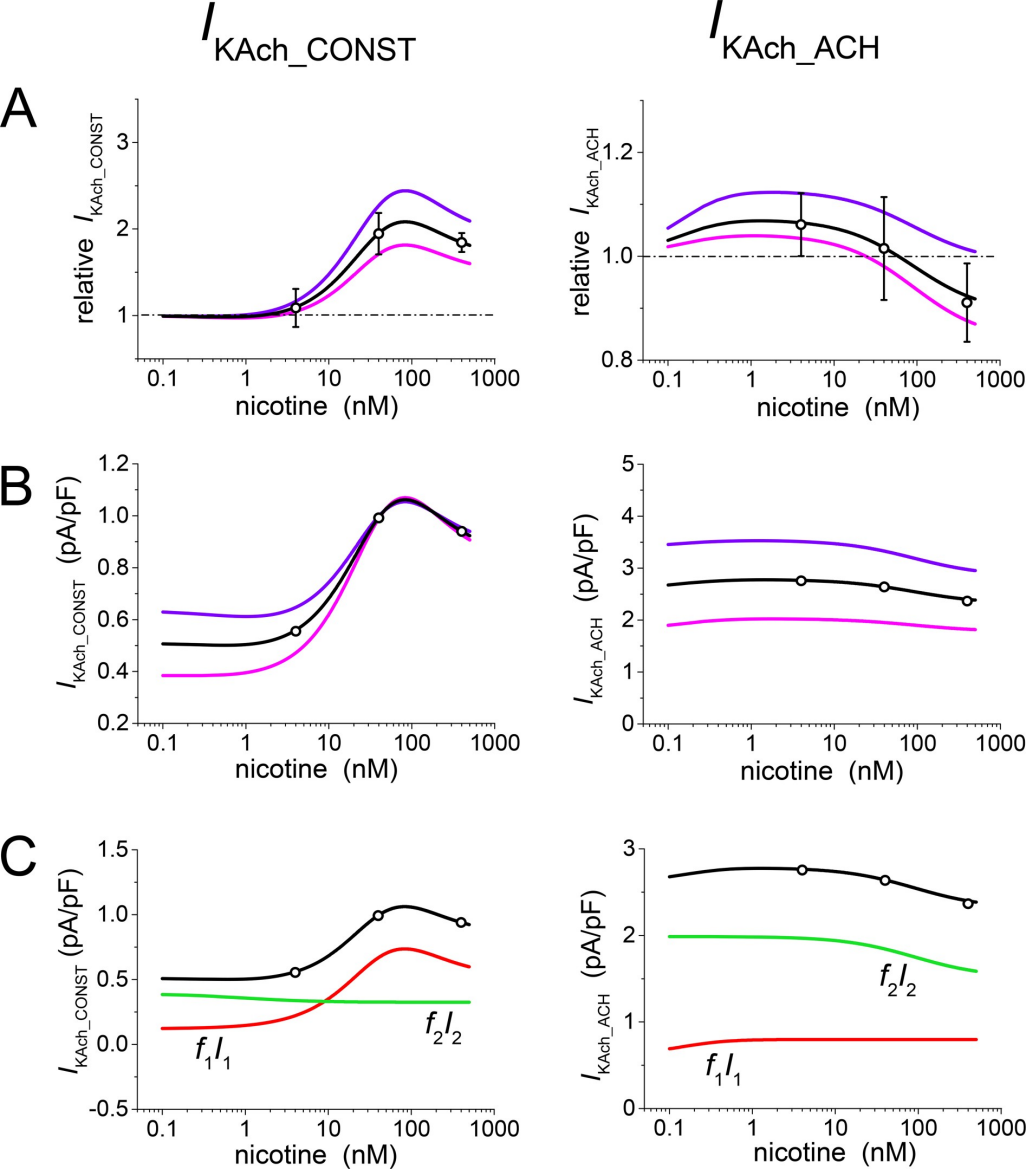
Analysis of the steady-state concentration dependence of the effect of nicotine on the constitutively active (*I*_KAch_CONST_) and acetylcholine-induced (*I*_KAch_ACH_) currents. Values of the parameters as summarized in Table 2 were used for calculations except for variations of *f*j needed to simulate dispersion of the measured currents. **A**: Relative changes of *I*_KAch_ components as functions of the nicotine concentration; black lines–model; circles–experimental data (± SE according to ref. [4]; 5–7 cells in the tested concentrations for *I*_KAch_CONST_ and 5–12 cells in the tested concentrations for *I*_KAch_ACH_). The data are related to the current densities at zero nicotine concentration. Simulations of the data dispersion result from small variations Δ*f* = 0.07 in fractions of individual populations of the *I*_KAch_ channels (*f*_1_+Δ*f*–blue lines, *f*_1_-Δ*f*–violet lines). **B:** Simulated *I*_KAch_ in absolute scale resulting from multiplication of the relative values (in part A) by the current densities at zero ethanol concentration (*I*_0_, Eq (5)). **C:** Contributions of the presumptive two current constituents (red and green lines) to the resulting nicotine concentration dependence of *I*_KAch_ at the basal setting of parameters according to Eq (4); *I*_*1*_ = *G*_1_ (*U-U*_K_), *I*_*2*_ = *G*_2_ (*U-U*_K_).

The effect of nicotine on both components of *I*_KAch_ in rat atrial myocytes (Fig 5) displayed main features similar to those displayed by the effect of ethanol (Fig 2). However, the voltage dependence of the effect was not negligible in the case of *I*_KAch_ACH_. Thus, one of the model parameters was considered as dependent on the membrane voltage. The effect of nicotine on *I*_KAch_ was analysed preferentially at –50 mV (i.e. in the range of positive currents) in contrast to the effect of ethanol which was measured mostly at -110 mV (negative currents). To reproduce experimental results measured at -50 mV in ref. [4], the values of the parameters summarized in Table 2 were inserted into the model. They were set the same way as in the case of ethanol, i.e. by repeated attempts to assure good fit with experimental data. The value of *f*_1_ related to drug-free conditions was left the same as in the above-described simulations of the ethanol effect (fixed to *f*_1_ = 0.68). In contrast, the values of parameters *h*_0_1_ and *h*_0_2_ had to be changed in simulations of the nicotine effect due to differences in the experimentally obtained current-voltage relations of the two separate sets of cells.

**Table 2:** Parameters of the model for simulations of the nicotine effect on *I*_KAch_.

Current	*f*_1_ -	*K*_1_ = *K*_2_ [mM]	*K*_3_ [mM]	*K*_4_ [mM]	*h*_0_1_ -	*h*_1_1_ -	*h*_2_1_ -	*h*_0_2_ _-_	*h*_1_2_ -	*h*_2_2_ -
*I*_KAch_CONST_	0.68	50	1	∞	0.008	0.107	0.036	0.06	0.049	0
*I*_KAch_ACH_	0.68	0.2	110	520	0.065	0.085	0.085	0.45	0.41	0.025

*f*_1_ *–*mean value of the first fraction of identical channel populations (*f*_2_ = 1- *f*_1_); *K*_1_, *K*_2,_ *K*_3_, *K*4 –drug dissociation constants; *h*k_j−dimensionless parameters related to the steady-state conductivities of the *j*^th^ channel population; *k* refers to occupation of binding sites.

The simulated concentration dependences of nicotine effect (Fig 5) are presented in the same way as those of ethanol (Fig 2). Relative changes in the experimental and simulated components of *I*_KAch_ were plotted against the nicotine concentration in Fig 5A. The steady-state effect of nicotine in *I*_KAch_ CONST_ (predominantly activation) was significantly stronger than the effect in *I*_KAch_ACH_. In the latter case, we even observed inhibition at 400 nM. After recalculation to absolute scale (Fig 5B), the currents have a positive sign at -50 mV. Fig 5C shows the resolution of *I*_KAch_CONST_ and *I*_KAch_ACH_ into constituents as predicted by the model.

**Fig 6:**
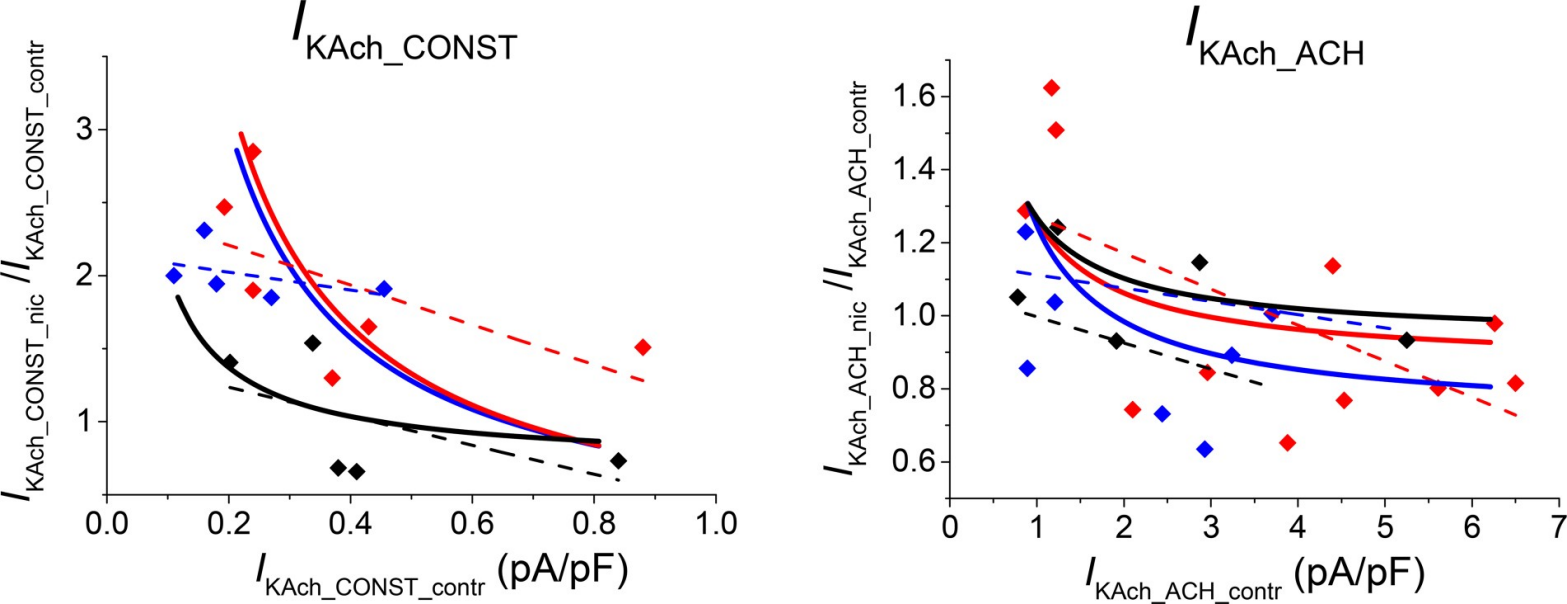
Correlation between the relative effect of nicotine (nic) on *I*_KAch_ components and the current density under nicotine-free conditions (contr). Relative *I*_KAch_CONST_ (left); relative *I*_KAch_ACH_ (right). Nicotine concentrations of 4, 40, and 400 nM (black, red, and blue lines and symbols, respectively; 5, 6 and 5 cells for *I*_KAch_CONST_ at 4, 40 and 400 nM nicotine, respectively; 5, 11 and 7 cells for *I*_KAch_ACH_ at 4, 40 and 400 nM nicotine, respectively). Symbols: experimental results from individual cells according to Bébarová *et al*. [4]. Dashed lines: linear regression analysis of the experimental data for each concentration separately. Full lines: simulated results using values of parameters as stated in Table 2. The variations of the current in control were simulated by continuous redistribution between the channel populations (fractions *f*_1_ and *f*_2_ = 1- *f*_1_).

As in the case of ethanol, the effect of nicotine correlated with the magnitude of *I*_KAch_ density in the absence of nicotine (at 4, 40, and 400 nM–Fig 6). The results of linear regression applied to the experimental data are plotted for all concentrations explored (dashed lines). The model simulations revealed nonlinear dependences (full lines).

**Fig 7:**
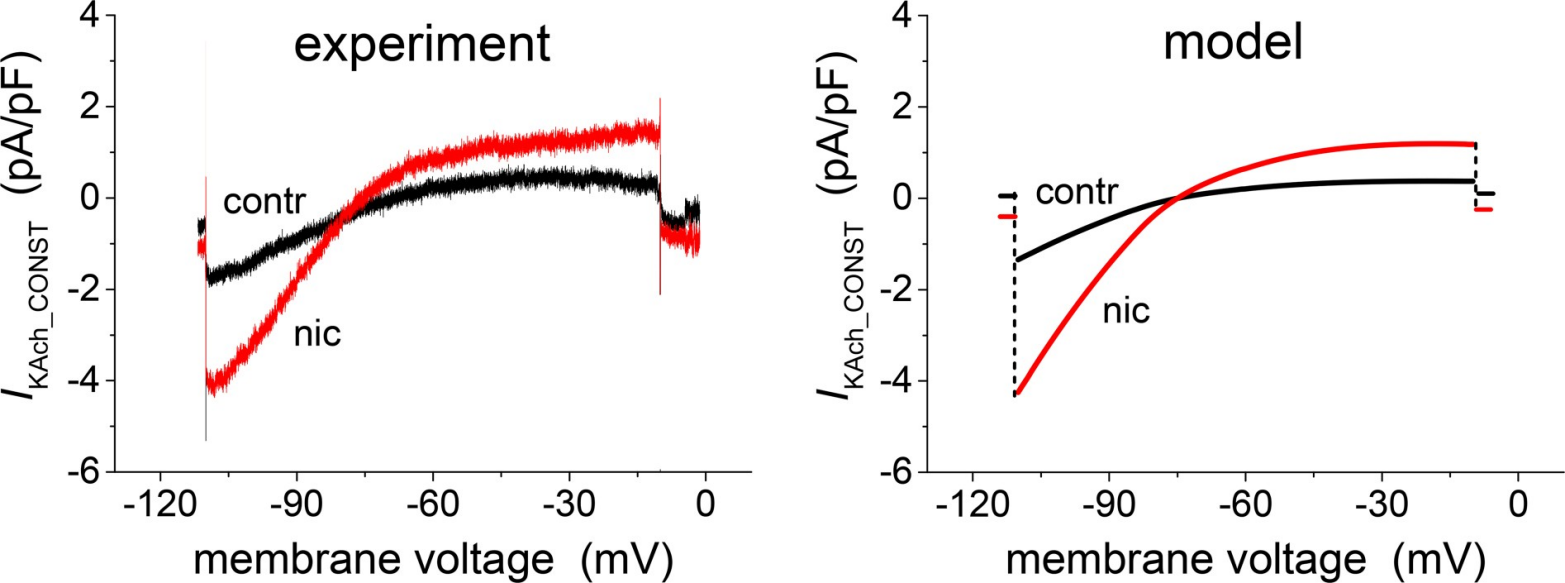
Effect of nicotine (40 nM) on the current-voltage relation of *I*_KAch_CONST_. Experimental record (left panel; 3 cells) and simulated data (right panel). Activation of the current was simulated by redistribution of the channels between fractions: (*f*_1_→ *f*_1_+Δ*f*, *f*_2_→ *f*_2_-Δ*f*, Δ*f =* 0.19).

The voltage dependence of the effect of nicotine was available only for *I*_KAch_CONST_ measured at a concentration of 40 nM (Fig 1E in ref. [4]). In Fig 7 model results are compared with experimental data. The nicotine effect was calculated as a result of an increase of the fraction of the channel *f*_1_ by Δ*f* = 0.19 on account of the same decrease in *f*_2_.

**Fig 8:**
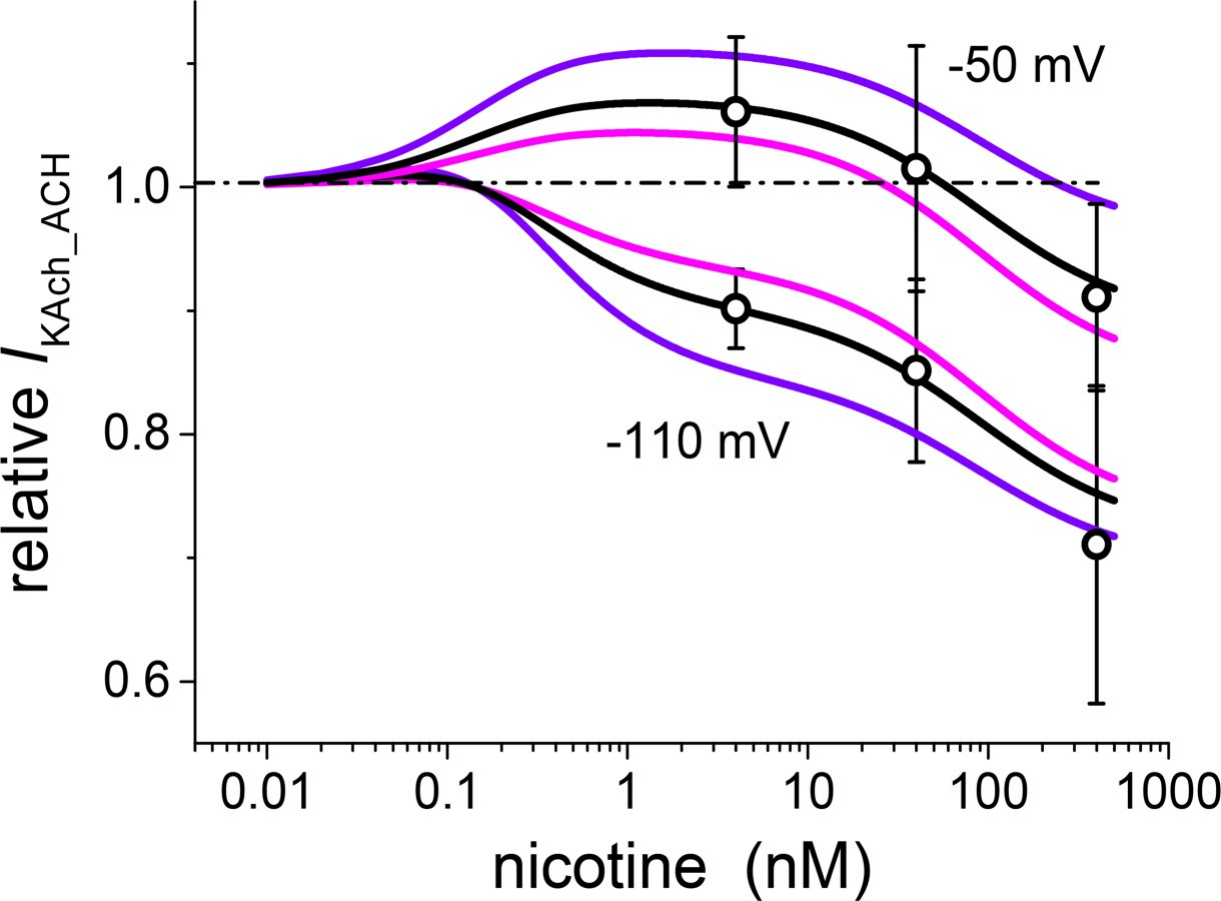
Steady-state concentration dependence of the effect of nicotine on *I*_KAch_ACH_ at -50 mV (above) and -110 mV (below) on a relative scale (currents under nicotine related to the respective control values; 5–12 cells in individual concentrations). The shift of the concentration dependence at -110 mV towards inhibition was simulated by a decrease of the activation related parameter *h*_2_1_ (from 0.085 to 0.037). All other parameters as summarized in Table 2 remained unaltered.

As mentioned above, the effect of nicotine was not strictly voltage independent. The concentration dependence of the relative drug effect on *I*_KAch_ACH_ measured at -110 mV was shifted towards inhibition in comparison with the results at -50 mV (Fig 3C in ref. [4]). The present model reproduced this shift (Fig 8) as a result of a decrease of the single parameter *h*_2_1_ related to activation.

## Discussion

The potassium inward rectifying channels are known to be composed of several different assemblies of α-subunits. These structural differences are manifested by various functional properties of the channels. However, to our knowledge, Kir currents carried by different populations of identical channels (i.e. sharing the same composition and ordering of α-subunits) have not been specified in the available integral models. While the channels responsible for *I*_K1_ in the ventricular and atrial cells are composed of three subunits (Kir2.1, Kir2.2 and Kir2.3), the *I*_KAch_ channels are homo- or heterotetramers comprised of two subunits (Kir3.1 and Kir3.4) (for review, see [9]). It has been well established that activity of the channels exhibiting inward rectification is modulated by a large variety of intracellular factors (e.g. PIP_2_, Na^+^, Mg^2+^, polyamines, pH and cAMP-dependent PK [9, 24–25]) and by various drugs [26–34], including anaesthetics and alcohols [6, 20, 23, 35–36].

Our recently published experimental studies focusing on the effects of ethanol and nicotine on Kir currents in cardiac cells [1–4] have shown that the interactions of these drugs with the Kir channels exhibited several characteristic features differing from the simple inhibition observed in the effect of ethanol on most other currents [37].

To quantitatively describe these unusual observations, we tried to develop a model that would reproduce available experimental results and suggest possible mechanisms underlying the drug effects. One version of our model respecting the molecular structure of the channels was tested on the observed ethanol-*I*_K1_ interaction in rat ventricular and atrial myocytes [10]. In the present study, the model is modified to reproduce the effects of ethanol and nicotine on the components of *I*_KAch_ (constitutively active *I*_KAch_CONST_ and acetylcholine induced *I*_KAch_ACH_) [3–4].

### Dual drug effect

A common feature of the effect of ethanol and nicotine on *I*_KAch_ (similarly to the effect of ethanol on *I*_K1_) is the unusual action of these drugs manifested by activation or inhibition at different drug concentrations (or in some cases at the same drug concentration in various cells) [1, 3–4]. *I*_KAch_CONST_ was only activated by ethanol and nicotine throughout the whole range of the investigated concentrations (Figs 2 and 5). However, *I*_KAch_ACH_ exhibited a slight inhibition at the lower ethanol concentrations, activation reaching a maximum at the medium concentrations, and a subsequent decrease of the activation effect (Fig 2). In the case of nicotine (Fig 5), the activation even reversed into an inhibition at the higher concentrations. Thus, it seems that the net effect of ethanol/nicotine modulation of Kir channels is determined by an alternant susceptibility to activation or inhibition.

These complex drug effects are interpreted in our model as resulting from two or more simpler processes related to the diversity of structures of the individual Kir channels formed by combination of the different α-subunits. Individual populations of identical channels are assumed to show different conductivities, and the total recorded whole cell current is described as a sum of *n* independent particular constituents exerting the drug-induced activation or inhibition. To reproduce experimental results, *n* = 3 was sufficient to simulate the ethanol-*I*_K1_ interaction. However, in the present study, we reduced the number of different channel population to *n* = 2, in agreement with the prevailing notion that the *I*_KAch_ channels are composed of two different α-subunits forming assemblies of homo- and heterotetramers GIRK4 and GIRK1/4. For simplicity, we have assigned the activation effect to one of the two populations and the inhibition effect to the other.

In experiments on expressed GIRK channels, Arial *et al*. [20] demonstrated that short-chain alcohols may be bound to hydrophobic pockets (formed by two adjacent subunits of the tetramer). These pockets, which are able to bind one or two ethanol molecules, are assumed to be responsible for ethanol activation of the GIRK channels. If the binding of a single molecule causes activation more effectively than the binding of two molecules, the interaction of ethanol with the pocket results in the biphasic concentration dependence (Fig 2C).

The alcohol-binding pocket is not involved in the alcohol-dependent inhibition because mutations in the pocket had no effect on the alcohol-dependent inhibition, suggesting that the inhibition was related to an alternate site. Surprisingly, a mutation in the GIRK4 pore-helix (GIRK4^S143T^) converted 1-butanol from an inhibitor to an activator (S3 Fig in ref. [20]).

It is worth mentioning that a dual effect similar to that described in this study was also demonstrated in the effect of the small molecule of halothane (volatile anaesthetic) on the GIRK channels expressed in *Xenopus oocytes* [5–6]. In line with our results, the constitutive active basal current was activated by halothane throughout the examined concentration range, while the acetylcholine-induced current was inhibited at lower concentrations of the drug, but activated at higher concentrations.

Interestingly, the dual effect of ethanol was also observed in the pentameric ligand-gated ion channels, consistent with a two-site model of the ethanol-induced activation and inhibition. Moreover, short-chain alcohols interacted with the excitatory site, while long-chain alcohols interacted with the inhibitory site [38–40].

### Similarities and differences in the effects of drugs on Kir currents

The relative changes induced by both ethanol (Fig 2) and nicotine (Fig 5) were considerably greater in *I*_KAch_CONST_ than in *I*_KAch_ACH_. Furthermore, while *I*_KAch_CONST_ was always activated, *I*_KAch_ACH_ was activated at moderate concentrations of both drugs and slightly inhibited at a low concentration of ethanol and high concentrations of nicotine.

The peculiar behaviour of the Kir3.x (GIRK) channels under the effect of ethanol has been explained by different affinities of these channels for phosphatidylinositol 4,5-bisphosphate (PIP_2_) in the absence and presence of acetylcholine [20,41]. All Kir channels are directly activated by phospholipid PIP_2_ with various affinities [25, 42–45]. It has been demonstrated that the channels with high affinity for PIP_2_ (such as Kir2.x channels that are essentially constitutively active) show high currents, but low sensitivity to activation by ethanol and other drugs [46]. The high affinity for PIP_2_ is also accompanied by a tendency toward drug-induced inhibition [20].

The Kir3.x (GIRK) channels in the absence of acetylcholine exhibit a low affinity for PIP_2,_ and are characterised by a low basal current with a high tendency to be activated by ethanol [20]. This corresponds to a small basal current (*I*_KAch_CONST_) and marked drug-induced current activation observed in rat atrial cells (Figs 2 and 5). Acetylcholine markedly increased the *I*_KAch_ (very likely due to the increased channel affinity for PIP_2_) and suppressed sensitivity to drug-induced activation. Concurrently, the inhibitory effects of ethanol at low concentrations (Fig 2) and of nicotine at high concentrations (Fig 5) were revealed. There is an analogy between the ethanol effect on *I*_KAch_ACH_ and *I*_K1_ [1]. In both cases the channels bind PIP_2_ strongly, and thus the basal current is high and shows a tendency to be partially inhibited at low concentrations of ethanol. Our model does not describe the role of PIP_2_ explicitly; however the impact of PIP_2_ is implicit in values of the parameters *h*_k_j_.

### Inverse correlation between *I*_KAch_ in control and the drug effect

An inverse correlation between the amplitude of *I*_KAch_ in the control conditions and the relative effect of drugs was another peculiar observation reproduced by the model simulations (Figs 3 and 6). In accord with the considerations in the previous section, the drug effect decreased with the absolute value of the control current. The low *I*_KAch_CONST_ currents were always activated, while the *I*_KAch_ACH_ currents (which were one order higher) were activated or inhibited at different drug concentrations. The model parameters were set primarily to reproduce the concentration dependences of the drug effect, including dispersion. The inverse correlation between the drug effect and *I*_KAch_ in control conditions was then also reproduced without readjusting the parameters, but only by varying the ratio of the fractions *f*_1_ */ f*_2_.

### Voltage dependence of the drug effects on *I*_KAch_

The presented model was formulated under the assumption that the drug effects were voltage independent. Consequently, the parameters in Tables 1 and 2 were regarded as voltage independent. In other words, the interaction of drugs with the mechanism responsible for inward rectification was assumed to be negligible. This condition was not fully satisfied in the case of nicotine. Compared with the relative effect of nicotine on *I*_KAch_ACH_ at -50 mV, the effect assessed at -110 mV was shifted towards inhibition at all applied concentrations (Fig 3C in [4]). A similar shift (between the effect at -110 and -40 mV) was observed under the effect of relatively large molecules of local anaesthetic bupivacaine (pure inhibitor) on GIRK1/4 channels [41]. In our model, simulation of the shift of relative concentration dependence at -110 mV against that at -50 mV (Fig 8) required a reduction of the single parameter (*h*_2_1_) responsible for the activation.

### Limitations of the model

The inwardly rectifying potassium currents are newly understood to be sums of individual constituents related to the families of identical channels characterized by the same structure. Although the model is based on the current knowledge of channel structure and function, the need for simple quantitative description did not allow for the capturing of detailed processes at the molecular level. In particular, the processes responsible for the voltage dependence (inward rectification) are not included, and the current-voltage relations are described by a formal mathematical expression. The model parameters are voltage-independent constants in order to comply with the observation that the ethanol effect itself appeared to be approximately voltage-independent. Nevertheless, one parameter had to be regarded voltage-dependent to simulate the impact of the voltage on concentration dependence of the nicotine effect (Fig 8).

The number of populations of identical channels is generally unknown. The value of *n* = 2 was chosen in agreement with the current view that the *I*_KAch_ channels in the atrial cells are restricted to two functional assemblies of α-subunits GIRK1/4 and GIRK4. Their specific assignment to the two channel populations in the model remains undetermined. It is unclear to what extent GIRK4 homo-tetramers versus GIRK1/4 hetero-tetramers dominate in cardiac cells [16]. The option of *f*_1_ = 0.68, i.e. *f*_1_/ *f*_2_ = 2.125 appeared to be the optimal mean value to display all the observed phenomena as a result of random variations of a single parameter *f*_1_/ *f*_2_. Simultaneously, the concentration dependence of the drug-effects, the observed scatter of the measured *I*_KAch_ values, and the dependence of the drug-effects on the baseline currents must be displayed. The observed scatter of the measured *I*_KAch_ currents is attributed to random variations of the *f*_1_/ *f*_2_ ratio according to the model; however, the proportion of the experimental error may not be negligible.

As mentioned above, the experimental data related to *I*_K1_ included transient changes of the current in response to the onset of ethanol. Corresponding transients in conductivities were described by Šimurda *et al*. in ref. [10] by differential equations. Drug-induced changes of *I*_KAch_ during transition to their steady-states have not yet been reported, and so such data is missing in this study. For possible future simulations, the model could be supplemented by the differential Eq (8). The conductivities *G*_1_ and *G*_2_ in Eq (3) refer to their steady-state values. Their transient changes at a given voltage in response to sudden drug application (designated here *G*_1,t_ and *G*_2,t_) are assumed to obey first-order differential equations

$$\frac{G_{1,t}}{dt}=\frac{1}{\tau_{1}}\left(G_{1,t}-G_{1}\right),\;\frac{G_{2,t}}{dt}=\frac{1}{\tau_{2}}\left(G_{2,t}-G_{2}\right),$$ (8)

where *τ*_1_ and *τ*_2_ are time constants. The corresponding time courses of the current in absolute and relative scale (*I*_t_ and *F*_t_) can be obtained if the quantities *G*_1_ and *G*_2_ in Eqs (4) and (6) are substituted by *G*_1,t_ and *G*_2,t_.

Localization of the binding sites for inhibition has not yet been fully elucidated. Assignment of activation to the first population (*f*_1_) and inhibition to the second population (*f*_2_) is more or less arbitrary. It is possible that each population includes both mechanisms with activation predominating in one and inhibition in the other. Their detailed incorporation into the model would unduly complicate mathematical description without bringing new insights into the problem. The number of parameters that should be fitted to the given experimental data would increase several times. Our main goal was to show that the mere fact that Kir channels are composed of populations with different functional properties is able to explain the complex drug-effects, not only in Kir2.x but also in Kir3.x channels.

## Conclusions

We conclude that the proposed model may serve as a useful tool for analysis and simulation of complicated manifestations of the interaction of some substances with the Kir channels. This approach is flexible and can be easily modified if new essential experimental findings become available. An analogical approach could be also used to describe other types of ionic channels—channels that do not show inward rectification but that may exhibit several possible channel populations with specific properties. Moreover, the model can be readily built into the existing integral models of electrical activity of cardiac cells in order to upgrade the description of Kir currents and their interaction with drugs.

## Materials and methods

### Experimental data

**Fig 9:**
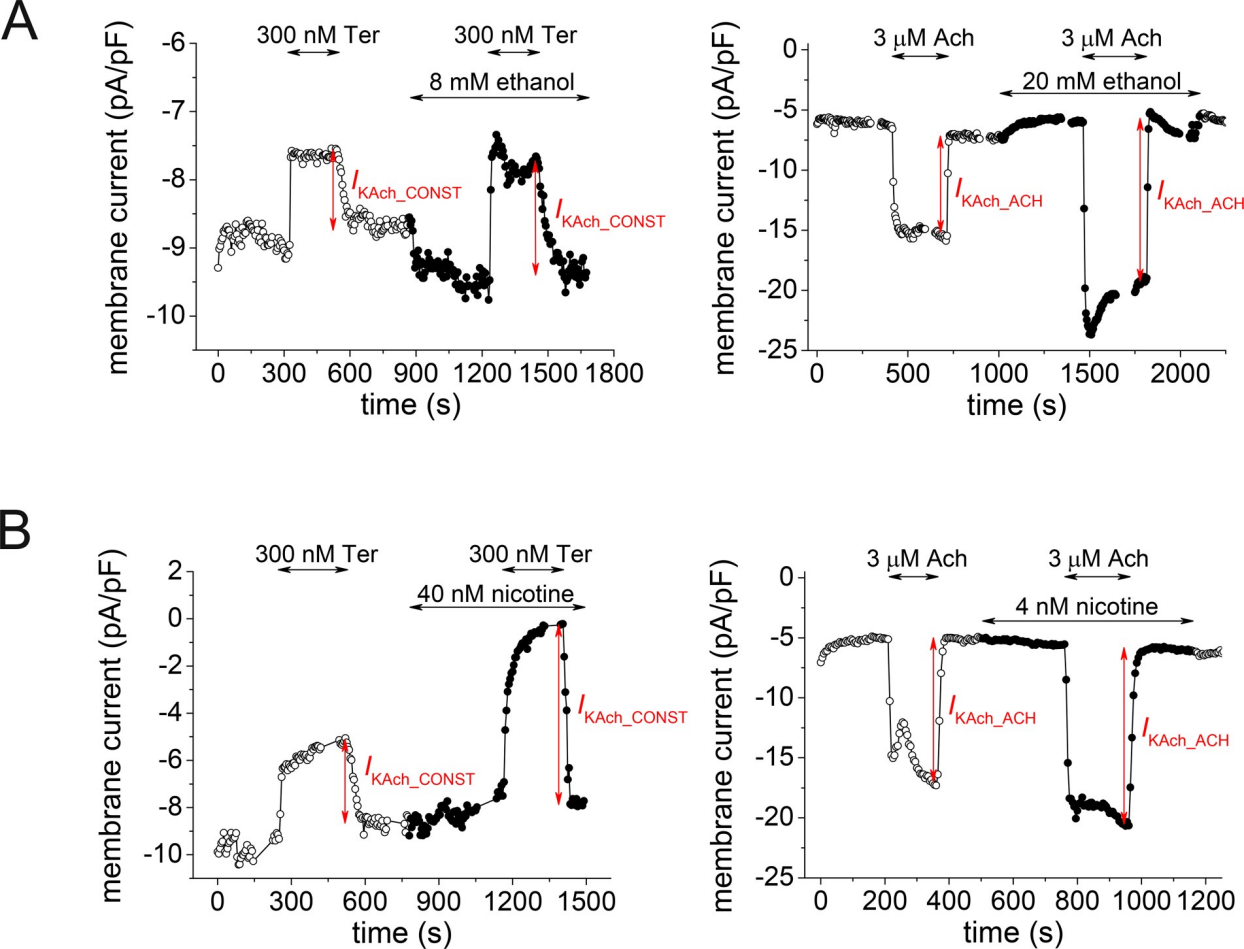
**Representative time courses of the membrane current density** during application of specific inhibitor of the *I*_KAch_ channels tertiapin (Ter; 300 nM) to reveal the constitutively active component of *I*_KAch_ (*I*_KAch_CONST_; left panels), or during application of acetylcholine (Ach; 3 μM) to activate the acetylcholine-activated component of *I*_KAch_ (*I*_KAch_ACH_; right panels), both in the absence and in the presence of ethanol (A) or nicotine (B) at selected concentrations.

The experimental data were obtained from our previously published studies [3–4], which give detailed information on the materials and methods. The constitutively active *I*_KAch_ (*I*_KAch_CONST_) was evaluated as the current sensitive to its specific inhibitor tertiapin-Q (300 nM) and *I*_KAch_ACH_ was evaluated as the current activated by the steady-state application of 3 μM acetylcholine (Fig 9). Both currents were measured at the end of a 500-ms rectangular pulse, from -85 mV to -110 or to -50 mV. To measure voltage dependence of the drug effects, 3-s ramp pulses between -110 and -10 mV were applied. In all experiments, the holding voltage was set to -85 mV and the stimulation frequency to 0.2 Hz. When necessary, the previously published data were recalculated to obtain the intended graphs (Fig 3 and Fig 6 left).

### Computer simulations

The Eqs (2)–(7), describing the effect of ethanol and nicotine on the rat atrial *I*_KAch_CONST_ and *I*_KAch_ACH_ [3–4] with values of parameters as defined in Tables 1 and 2, were solved numerically using the computational software MATLAB v. 7.2 (MathWorks, Inc.). The MATLAB files for modelling of ethanol and nicotine effects on *I*_KAch_ are available from authors upon request.

## Abbreviations

c: Drug concentration
*f*_j_: Generally: *j*^th^-fraction of identical channel populations
*f*_1,_*f*_2_: 1^st^ and 2^nd^ fraction of identical channel populations for *I*_KAch_
Δ*f*: Random shifts between fractions (*f*_1_ +Δ*f*, *f*_2_ *-*Δ*f*
F: Relative current (*I/I*_o_)
*g*(*U*): Approximation of the voltage-dependent conductivity of *I*_KAch_
*G*_1,t_, *G*_2,t_: Conductivity of the 1^st^ and 2^nd^ channel population
*G*_1_, *G*_2_: Steady-state conductivity of the 1^st^ and 2^nd^ channel population
*G*_k_1,_*G*_k_2_: Partial steady-state conductivities of the 1^st^ and 2^nd^ channel population related to the drug occupation of binding sites (*k* = 0, 1 or 2)
GIRK: G-protein modulated inwardly rectifying potassium channels (GIRK4, GIRK 1/4)
*h*_k_1_, *h*_k_2_: Dimensionless parameters (*G*_k_1_ = *h*_k_1_ *g*(*U*), *G*_k_2_ = *h*_k_2_ *g*(*U*))
*I*, *I*_Kir_: Inward rectifier potassium current (generally)
*I*_j_, *I*_Kir_j_: Constituents of *I* (*I*_Kir_) related to individual populations (*j*) of identical channels
*I*_K1_: Inward rectifier potassium current Kir2.x
*I*_KAch_: Acetylcholine-sensitive inward rectifier potassium current (Kir3.x)
*I*_KAch,0_: Acetylcholine-sensitive inward rectifier potassium current under drug free conditions
*I*_KAch_CONST_: Constitutively active component of *I*_KAch_
*I*_KAch_ACH_: Acetylcholine-induced component of *I*_KAch_
I, I: Components
j: Subscript related to individual populations of identical channels (generally *j* = 1, …, n)
k: Subscript related to occupation of binding sites
*K*_1_, *K*_2,_*K*_3_, *K*_4_: Dissociation constants
n: Number of populations of identical channels (*n* = 2 for *I*_KAch_)
PIP_2_: Phospholipid phosphatidylinositol-4,5-bisphosphate
*τ*_1,_*τ*_1_: Time constants
U: Membrane voltage
*U*_K_: Equilibrium voltage for potassium ions
*x*_k_j_: Probability of channels to be found in state *k* pertaining to *j*^th^ channel population
